# Potential worldwide distribution of Fusarium dry root rot in common beans based on the optimal environment for disease occurrence

**DOI:** 10.1371/journal.pone.0187770

**Published:** 2017-11-06

**Authors:** Renan Macedo, Lilian Patrícia Sales, Fernanda Yoshida, Lidianne Lemes Silva-Abud, Murillo Lobo

**Affiliations:** 1 Universidade Federal de Goiás, Escola de Agronomia e Engenharia de Alimentos, Goiânia, GO, Brazil; 2 Universidade Federal de Goiás, Departamento de Ecologia e Evolução, Goiânia, GO, Brazil; 3 Empresa Brasileira de Pesquisa Agropecuária–Embrapa Arroz e Feijão, Santo Antônio de Goiás, GO, Brazil; Universita degli Studi di Pisa, ITALY

## Abstract

Root rots are a constraint for staple food crops and a long-lasting food security problem worldwide. In common beans, yield losses originating from root damage are frequently attributed to dry root rot, a disease caused by the *Fusarium solani* species complex. The aim of this study was to model the current potential distribution of common bean dry root rot on a global scale and to project changes based on future expectations of climate change. Our approach used a spatial proxy of the field disease occurrence, instead of solely the pathogen distribution. We modeled the pathogen environmental requirements in locations where *in-situ* inoculum density seems ideal for disease manifestation. A dataset of 2,311 soil samples from commercial farms assessed from 2002 to 2015 allowed us to evaluate the environmental conditions associated with the pathogen’s optimum inoculum density for disease occurrence, using a lower threshold as a spatial proxy. We encompassed not only the optimal conditions for disease occurrence but also the optimal pathogen’s density required for host infection. An intermediate inoculum density of the pathogen was the best disease proxy, suggesting density-dependent mechanisms on host infection. We found a strong convergence on the environmental requirements of both the host and the disease development in tropical areas, mostly in Brazil, Central America, and African countries. Precipitation and temperature variables were important for explaining the disease occurrence (from 17.63% to 43.84%). Climate change will probably move the disease toward cooler regions, which in Brazil are more representative of small-scale farming, although an overall shrink in total area (from 48% to 49% in 2050 and 26% to 41% in 2070) was also predicted. Understanding pathogen distribution and disease risks in an evolutionary context will therefore support breeding for resistance programs and strategies for dry root rot management in common beans.

## Introduction

The spatiotemporal distribution of plant diseases follow the changes undergone by agriculture, attributed to climatic variation and technological shifts. Large monocultures are predominantly subjected to disease outbreaks or frequent disease occurrences, thus usually relying on high-input technologies to sustain production and avoid yield losses. In these circumstances, maps of disease risks are therefore safeguarded to prevent large-scale outbreaks or relevant problems with crops and food security threats, supporting crop management in regular, well-defined climatic conditions [1,2]. However, the dynamic nature of plant diseases brings new uncertainties to crop management and to breeding programs required to ensure yields in the future [3].

The climate requirements of soilborne pathogens, such as temperature and precipitation [4], define disease favorability in specific regions where the host crop is present, and determine the array of practices required for disease management. The unbalance in well-defined requirements for disease occurrence [3,4] by the predicted changes in climate around the world will therefore potentially change disease distribution and disease management in several crops [5,6]. Therefore, overall disease modelling should be concerned about these complex interactions that affect pathosystems, in order to estimate pathogen spread and disease risks in different scenarios [7]. If we aim to prevent yield losses and improve food security, disease scouting is crucial to adjust research and development efforts that will enhance disease management, supporting decisions at the farm level and public policies [2].

Potential distribution models are commonly used in conservation biology and other research topics to predict species distribution through climate (environmental) requirements [8]. They are correlational approaches based on how environmental constraints may limit the occurrence of specific “objects”—usually species or populations—at broad spatial scales [9]. By modelling the environmental conditions associated with an object’s occurrence or abundance, spatial projections of favorability can therefore be assessed. Such models highlight potentially suitable regions for the event of interest, in the current scenario or in climate change forecasts [8]. Although this framework has traditionally been used in conservation-related research, it conceivably may be useful in plant disease epidemiology studies, with macro-ecological tools [7].

It is possible to estimate both pathogen and disease geographical distribution because both objects may coexist in regions where the climate is more or less favorable for them. Regarding crop protection, potential distribution maps have been used to anticipate risks for vector-borne plant diseases [10] and insect pests using species distribution models [11]. Thus, models of potential distribution may shed light on unanswered epidemiological questions concerning the spatial distribution of diseases, and the shifts in the intensity of disease episodes expected with climate changes [8].

Disease scouting and risk mapping are especially important for staple foods, such as common beans (*Phaseolus vulgaris* L.), an essential protein source for developing countries in Latin America and Africa [12]. Soilborne pathogens, such as the *Fusarium solani* species complex (FSSC), regularly challenge common bean crops [13,14]. Broadly distributed in the world, the FSSC is a generalist pathogen that is well adapted to a wide array of environmental conditions and hosts [15]. The FSSC causes root rot and yield losses up to 100% [16], leading to chronically lower yields in most regions [13].

In this study, we used the inoculum threshold approach represented by inoculum density, a highly used measure for epidemiological studies [17], easily estimated in field-soil samples. Although widely distributed in tropical regions of South America and Africa [18], no consensus has been reached on the lower inoculum threshold associated with dry root rot occurrence in common beans. Moreover, the link between FSSC spatial distribution and dry root rot records has not yet been spatiality investigated. Here, we have provided the first attempt to model the worldwide geographical distribution of common bean dry root rot. Using an innovative approach, we speculated if the propagule optimal threshold of the FSSC could be used as a proxy of dry root rot occurrence in common beans in Brazil. That spatial proxy of the disease occurrence was then examined to model its worldwide distribution. We therefore tested the following hypotheses: 1) An optimal inoculum density threshold is spatially linked to disease occurrence; 2) root rot risk areas coincide with the main regions for common bean cropping; and 3) climate change will affect the current disease distribution.

## Materials and methods

In this study, we modeled the potential distribution of common bean dry root rot, via species distribution model/ecological niche model procedures, which required field records of the disease, referred to as the object of interest. Precise, geo-referenced records for dry root rot in common beans are usually lacking. Governmental disease reports usually provide information solely on the state or municipality where a disease was detected, from which GIS-based information, i.e., coordinate references, cannot be accurately derived. To overcome such a limitation, we derived a method for refining our disease occurrence dataset, by using a reliable spatial dataset on the inoculum density of common bean dry root rot. We used the FSSC inoculum density that was most spatially correlated to disease occurrence (explained below), assuming that the common bean is mostly a highly susceptible crop and that all isolates from the main Brazilian common bean growing regions are pathogenic [19].

### Spatial proxy calculation

The inoculum density of soilborne pathogens is dependent on the climate and cropping system [14]. Even though some authors report favorable temperatures for the disease (*e*.*g*. above 18°C) [14](*e*.*g*. between 22–32°C) [18], and growth, survival and chlamydospore germination, the adaptation to climatic variations in both temperate and tropical regions, turns the FSSC into a cosmopolitan pathogen [15].Growing drivers, such as the temperature and soil moisture, coupled with the nutrients and organic matter content in soil, directly affect the viability of chlamydospores (the resistance structures of several *Fusarium* species) and the seasonality of the FSSC [20]. The same variables affect directly host root development in different cropping systems [14].

FSSC chlamydospores are broadly distributed in soil throughout the year, and growing hyphae exhibit great saprophytic ability [21]. In this study, a database of FSSC inoculum density records managed by Embrapa Arroz e Feijão (Santo Antônio de Goiás, Brazil) supported the pathogen spatial proxy estimate. That database was composed of 2,311 soil samples from commercial farms that belonged to 103 municipalities in 10 Brazilian states assessed from 2002 to 2015. Common beans were always present in the cropping history of sampling sites. In general, samples were taken at 0–10 cm topsoil from commercial farms where common bean-maize-soybean is the main cropping sequence, chosen by farmers due to commercial demands. Eventually, cropping sequences included sorghum, millet, vegetables, or forage crops. Each soil sample was submitted to serial dilution and deep plating in Nash-Snyder semi-selective medium. The FSSC colonies were identified and their colony forming units (CFUs) per soil gram considered to estimate FSSC inoculum density. Former strain pathogenicity tests with the inoculum layer method [22] showed that all isolates caused average or high dry root rot severity to the common bean [19], supporting the modeling studies with feasible field records of inoculum density (S1 Table).

Disease data were retrieved from publications with field records of dry root rot on common bean crops. Scientific papers, short communications, and technical documents were selected according to the infestation histories of the sampling sites. Only those publications for which experiments had been conducted in sites naturally infested with the FSSC or in sites with historical records of dry root rot were kept. Ten publications sufficiently met the selection requisites and the geographic coordinates of their experimental sites, and therefore, were registered to support 46 disease occurrences (S1 Table).

To find the best spatial proxy for the disease, with a reliable spatial reference, we estimated the Spearman’s correlation coefficient between the disease occurrence and FSSC inoculum density records. Three inoculum densities were selected according to published data regarding the disease severity/inoculum density relationship. The inoculum densities adopted were the following: 1200 CFU/g of soil based on field experiments and controlled environment tests with the common bean and FSSC [23]; 3700 CFU/g of soil in greenhouse experiments with the same pathosystem as above [24]; and 4500 CFU/g of soil based on greenhouse tests to estimate the dry root rot severity according to increasing concentrations of FSSC chlamydospores [17]. The inoculum density that most correlated with dry root rot records was then considered a spatial proxy for the disease.

### Climate and climate change information

Climate data, used here to calibrate models of potential distribution, were downloaded from the WorldClim website (www.worldclim.org/current). This database was produced via the interpolation of data from ground weather stations referring to the years of 1950–2000 [25]. On WorldClim, climate data are available as raster files containing grid-based information at different spatial resolutions. We upscaled the downloaded rasters to the resolution of 0.5 degrees of lat/long. Although our inoculum dataset allowed a finer resolution analysis to be conducted, we opted for a relatively coarser spatial resolution. Because the exact location of the sampling sites was not known, the resolution of spatial data on disease occurrence (extracted from publications) was usually rough. Several papers indicated only the municipality and not the exact locations (and the associated environmental conditions) of the experimental sites. However, most municipalities to which studies usually referred were smaller than 50km^2^. Therefore, we assumed that a 0.5 lat/long degree could adequately encompass the average enviromental conditions associated with all of the possible locations where experiments could have been conducted. In this way, the loss of some important local environmental information was compensated for by more accurate patterns on a worldwide scale, due to more generalized relationships between climate and disease occurrence.

In addition to estimating the potential distribution of dry root rot in the present- day, we also evaluated the potential impacts of climate change on disease spread. To do so, we projected the disease distribution models into future climate scenarios. Scenarios of climate change were taken from two “Representative Concentration Pathways”—RCPs—from the Intergovernmental Panel on Climate Change (IPCC). Each RCP is based on a greenhouse gas concentration trajectory that the IPCC adopted for its Fifth Assessment Report (AR5) [26]. The RCP 2.6 estimates an increase of global warming of 1°C by 2050 (average for 2041–2060), whereas the RCP 8.5 projects an increase of 2°C. The same scenarios are used for 2070 (average for 2061–2080) with the RCP 2.6 (1°C) and the RCP 8.5 (3.7°C) [26]. Each RCP scenario is calculated from monthly temperature and precipitation averages, which the climate forecasts known as Atmospheric and Oceanic General Circulation Models (AOGCMs) generated [27]. Bioclimatic variables therefore are calculated from monthly temperature and precipitation values, representing annual climatic trends, seasonality, and extremes. Those climate parameters are usually considered environmental constraints for biological species and systems [28].

Although AOGCMs have been widely used to project the impacts of climate change on species distribution, different forecasts may provide different outcomes [27]. Variation in the AOGCMs chosen to project distribution models for the future can thus affect maps of potential distribution, creating a well-known source of uncertainty [29]. We therefore accounted for that known source of uncertainty by selecting different AOGCMs and projecting the disease distribution models onto all of them. The following climate forecasts were chosen: the Community Climate System Model (CCSM4) version 4 [30], the Hadley Centre Global Environmental Model version 2 (HADGEM2) [31], and the Model for Interdisciplinary Research on Climate (MIROC5) [32].

These climate forecasts are based on robust modelling algorithms, although each one has a different bias in terms of temperature and precipitation estimates [27]. The CCSM4, for example, has biases in average precipitation distribution in the tropical Pacific Ocean [30], and the HADGEM2 shows some bias toward warmer temperatures in the continental Northern Hemisphere and a colder bias in South America [31]. Lastly, MIROC5 also shows a cooling bias in the North Atlantic [32]. Therefore, no AOGCM is efficient in everything [27]. Such biases may, however, be ameliorated by considering different AOGCMs as a source of variation in model outcomes to improve the predictions of distribution models [33].

Climate forecasts provide predictions for several environmental variables based on temperature and precipitation—the bioclimatic variables. However, not all of these predictors are used in modelling procedures, to avoid correlation and collinearity-related problems. We therefore selected the predictors to be used in modelling procedures, by using logistic regressions. Logistic regressions are meant here to evaluate which bioclimatic variables would be the best disease predictors by calculating slopes without or with low collinearity and, at the same time, showing satisfactory biological explanations. All bioclimatic variables were considered as predictors. The response variable in such logistic regressions was disease occurrence (presence or absence), defined by the best spatial proxy, which was described earlier in the text. Logistic regression was performed with the R function *glm*, the binomial family, and the *logit* link. The binomial family is adequate for presence-absence data, and the *logit* link is the natural log of the odds in which the response variable equals one of the categories (zero and one categories). Non-significant predictor variables were removed. Further, collinearity between predictor variables was avoided with a cutoff on Pearson’s correlation coefficient (*r* < 0.25), which is considered statistically significant (*p* < 0.05) [34]. Those procedures led to the following bioclimatic variables, to be used as predictors in subsequent distribution models: Isothermality (mean diurnal range/temperature annual range), maximum temperature of the warmest month, precipitation seasonality (coefficient of variation), and precipitation of the warmest quarter.

The bioclimatic variables were therefore selected to allow appropriate statistical analyses. However, biological and ecological reasons also exist for justifying their choice as the best climate predictors [35]. Two chosen bioclimatic variables are based on the maximum and seasonal temperature, which admittedly alters the occurrence of diaseases. The precipitation seasonality is also a known driver of soilborne pathogen growth [4]. The rationale of modelling disease distribution based on optimal temperature ranges may not be efficient for predicting where disease does not occur [35]. Climatic constraints (maximum and minimum) may, however, lead to insights on the growth, development, and fitness of species over a period of time [35]. Periods of drought and rain in regions that exhibit high climatic seasonality may therefore influence the FSSC. These climate constraints [36,37] are indeed known to affect chlamydospore and conidia germination, spore viability, and agressiveness in different cropping systems [14].

### Distribution models for the *Fusarium solani* species complex

In this work, the study model comprised a soilborne pathogen (FSSC) and a host (common bean), used here to assess dry root rot distribution. As explained before, we used a spatial proxy defined by the inoculum threshold that best represented disease occurrence. Therefore, we modeled the pathogen environmental requirements found at locations where *in-situ* inoculum density seems ideal for disease manifestation. The environmental requirements of a pathogen are usually linked to disease occurrence via conditions (abiotic factors) and resources (biotic factors, which may be consumed and are subjected to competition) required for the pathogen’s survival and breed [38]. However, models based solely on ecological niches of pathogens may not lead to accurate maps of disease risk because the presence of a pathogen does not necessarily implicate in disease manifestation [39]. Thus, by using not only the pathogen presence but also the density most correlated to disease manifestation, we modeled the environmental conditions most strongly related to the dry root rot occurrence. The resulting host × pathogen × environment interaction is therefore the pathogen niche projected for disease occurrence and represented in maps of climatic suitability of dry rot root in common beans.

Common bean crops account for about 3.1 million hectares cropped in all Brazilian regions, especially in the South, Southeast, and Center-West regions, which themselves are responsible for 3,327.8 thousand tons of the crop [40]. In Brazil, diseased fields are frequently reported, especially due to conducive weather and the omnipresence of susceptible cultivars [41]. In this study, models of FSSC distribution were calibrated with data retrieved for the Brazilian territory extent, although projections were made worldwide. We did so because the access to the exclusive database on inoculum densities allowed us to characterize regions in the pathogen’s climatic niche, e.g., the environmental preferences and constraints [42–44], in a way not repeatable with other countries’ datasets.

By calibrating our distribution models exclusively with Brazilian data, we therefore assume that the relationship among the disease occurrence, inoculum density, and environmental requirements can be extrapolated to other regions with similar climatic conditions. This approach, although possibly new in plant disease epidemiology, is widely used in other niche-related areas, such as biological invasion risk assessments [45]. By using such an approach, our attempt here is to provide a first visual map of the dry root rot potential distribution worldwide, but not a guide for supporting local farmers and crop managers. Our models probably do not capture local climate peculiarities due to territorial data restrictions, even though disease projections may match disease records elsewhere. Therefore, we caution that the world maps provided here should not be used beyond their intended purpose.

Another known source of uncertainty in distribution models, beyond AOGCM climate forecasts, is the modelling method used to establish the relationship between the occurrence of the object of interest and the environment it occupies. Different modelling methods may provide dramatically distinct projections [8]. To minimize such discrepancies, we considered only a few different methods and weighted their projections according to their performances. That model-weighting procedure, also called “model ensembling”, assumes that by encompassing different possible projections and their respective performances, uncertainty in the modelling method is therefore minimized [46]. Such ensembles should not, however, encompass all classes of modelling methods, as they may have different requirements for model building and thus non-comparable results [47]. We therefore accounted for model uncertainty by ensembling among projections from different methods but also respecting theoretical restrictions regarding outcome comparisons.

Our ensembles of distribution models were performed within two main classes of models: 1) the statistical methods and 2) the machine-learning methods. No ensemble was performed between both classes of methods. Statistical or regression-based methods (e.g., a generalized linear model [GLM], a generalized additive models [GAM], and multivariate adaptive regression splines [MARS] allow for encompassing a large number of relationships between occurrences and environmental factors and usually have good explanatory power regarding the distribution-related ecological processes [47]. In this class of methods, precision and generality are balanced, which leads to moderately flexible although accurate predictions [48]. Machine-learning methods, in contrast, employ data-mining algorithms—such as GARP (Genetic Algorithm for Rule Set Production), random forest, and artificial neural networks—in an attempt to maximize the relationship between environmental predictors and biological responses [47]. Machine-learning methods usually lead to highly accurate but more clinger distribution predictions (rigid predictions) [48]. In machine-learning methods, generality is penalized for the sake of accuracy, and model complexity usually prevents a clear interpretation of parameter relationships [47].

The regression-based methods used in this work were a GAM and a GLM because they are compatible in building requirements [48]. The GAM was implemented with the function of *gam* using the binomial family and selecting 10,000 pseudo-absences randomly sampled from all occurrence points within our defined extent. Meanwhile, the GLM was adjusted with a linear function using a binomial family, being performed for stepwise proceeding, and using 10,000 pseudo-absences with random sampling, to avoid collinearity issues [48]. All analyses were performed in “sp” [49], “raster” [50], and “Biomod2” [51] packages within the R environment (R Development Core Team).

The machine-learning methods used in this study were the random forest and classification tree analysis. Random forest is a general technique of random decision forests. Here, we used 1,000 pseudo-absences collected with a random selection of points outside of the suitable area estimated with a rectilinear surface envelope from the large presence number (surface range envelope model “SRE”) [48]. Finally, we performed a classification tree analysis (CTA) with the same random forest framework described above, to assure model outcome compatibility. All models were fitted with 100 runs.

In a data-splitting process, 75% of our occurrence data were used for training and 25% for testing the model performance [48]. Model weighting was based on the True Skill Statistics (TSS), a measure of model performance that the prevalence of occurrences does not affect [52]. Sensitivity is the probability that the model correctly rates the presence of data, and specificity is the probability that it correctly rates an absent data. Values of TSS (TSS = sensitivity + specificity -1) range from -1 to +1, where values close to +1 indicate high accuracy, whereas values equal to or smaller than zero are usually considered not better than random. Less biased than other criteria, the TSS is the measure of choice for distribution predictions [53]. We used a cutoff (TSS < 0.5) to select exclusively the models with the best accuracy, i.e., models with TSS smaller than this figure were removed and not considered in posterior weighting procedures. Therefore, a weight (the TSS value) was attributed to each cell-based prediction of environmental suitability, which resulted in an averaged ensemble for each modelling method.

Ensemble models were projected onto current climate maps to provide estimates of potential distribution for the FSSC. The same ensemble models were then projected onto predictions of future climate forecasts, which were previously collected. Therefore, suitable regions were identified for disease distribution in the present-day time and in different scenarios for climate change. Projections were made on both a country (Brazil) and a worldwide scale to predict the disease risks of dry root rot on growing regions of common beans elsewhere [54]. All of the modelling procedures were performed with the R “Biomod2” package in R (R Development Core Team).

### Uncertainty analysis

Despite considering different projections, i.e., the ensembling procedure, we were also interested in determining which were the main drivers of total uncertainty in the model outcomes. To disentangle the variation present in our results, we adjusted linear mixed models with residual maximum-likelihood estimation (REML). Prior to that, we performed an estimability test of the effects in the mixed model in R [55] to check if it could be used to correctly describe factor rankings. We therefore built the mixed model considering the “year” (year 2050 and year 2070) as a random factor and considering “AOGCMS,” “RCPs,” and “modelling methods” as fixed factors. The Gauss-Markov function of the parameters was considered to be estimable if built by a linear combination of mathematic expectations. The best empirical linear unbiased estimates (eBLUEs) for fixed factors and the empirical best linear unbiased predictors (eBLUPs) were also performed for random factors. Thus, we separated the percentage of variation attributed to each factor. Because our model outcomes are maps, uncertainty was calculated for each grid cell. Therefore, uncertainty maps could be obtained for total variation and for the variation attributed to each factor. All maps presented in this paper are original and were created in R and ArcGIS 10.1 (ESRI, Redlands, CA, USA).

## Results

All three inoculum thresholds of the FSSC per soil gram showed correlation with disease occurrence: 1200 propagule/ soil gram (*ρ =* 0.79; p = 0.001), 3700 propagule/ soil gram (*ρ =* 0.85; p = 0.001) and 4500 propagule/soil gram (*ρ =* 0.81; p = 0.001). Therefore, the best correlation between propagules density and root rot occurrence in the common bean was 3700, as a lower threshold, considering the current disease spatial distribution. The 3700 lower threshold was therefore chosen as a proxy of the occurrence of common bean dry rot root in distribution models (78 occurrences). Indeed, the area of the disease distribution varied according to the proxy chosen: between 3700 propagules/soil gram and 1200 propagules/ soil gram the area reduced 3% and between 4500 propagules/ soil gram and 3700 propagules/soil gram the area reduced 21%. We projected also the distribution of the disease using the three proxies to 2050 and 2070, by the statistical method. We observed that to 2050, between the two RCPs, there was no a pattern of the disease distribution–an increasing or decreasing of its range with the increase of average temperature between RCPs. However, to 2070 we found the following pattern: the increasing of the inoculum threshold (from 1200 to 4500) is associated to an enhanced shrink of the disease distribution (S2 Table, S1 Fig).

**Fig 1:**
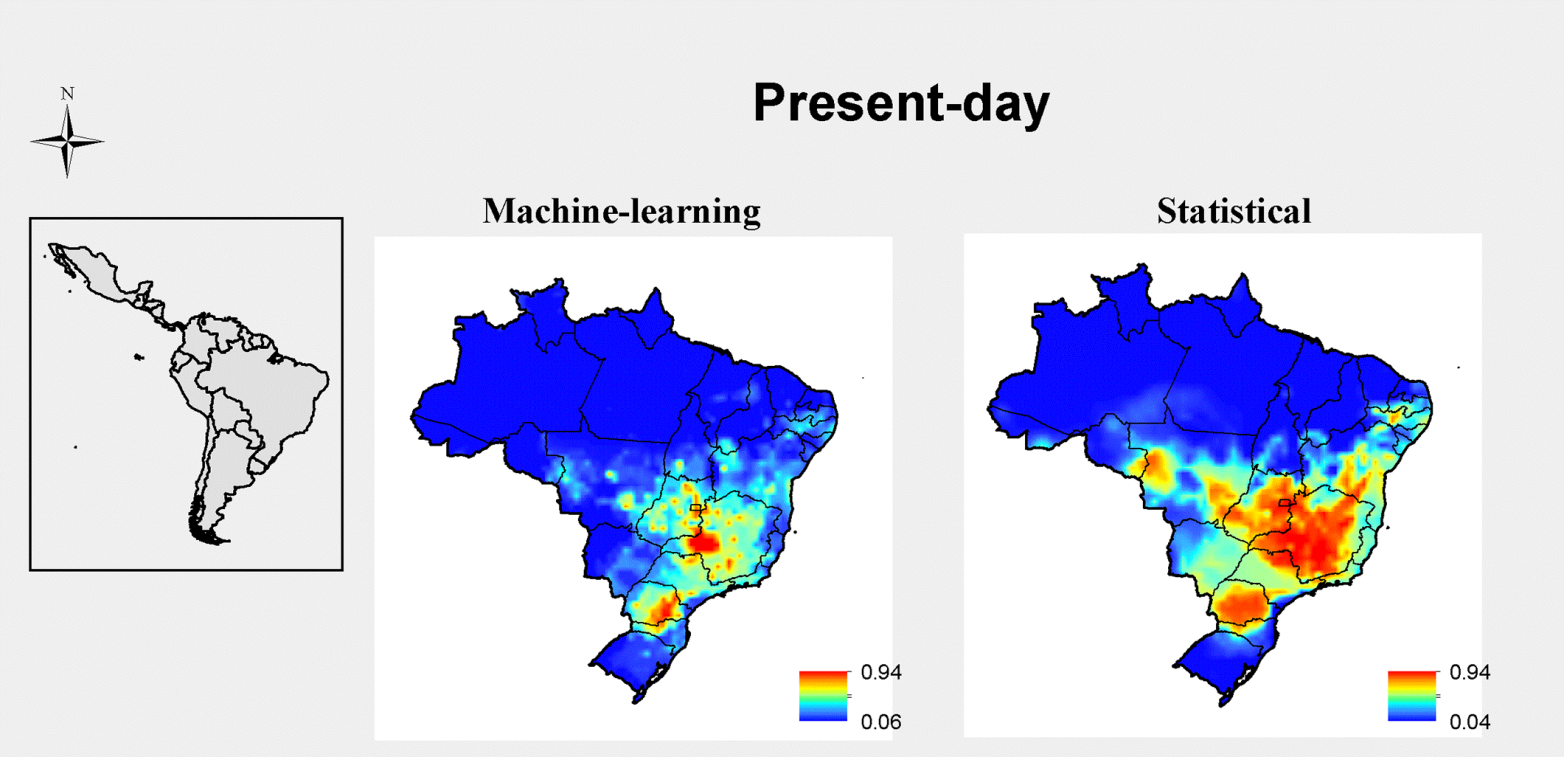
Current distribution of the FSSC, considering an inoculum density (3700 propagules per soil gram) in common bean crops as a proxy of disease occurrence in Brazil. The legend shows the climatic suitability: 1 is the most adequate, and 0 is the least suitable.

**Fig 2:**
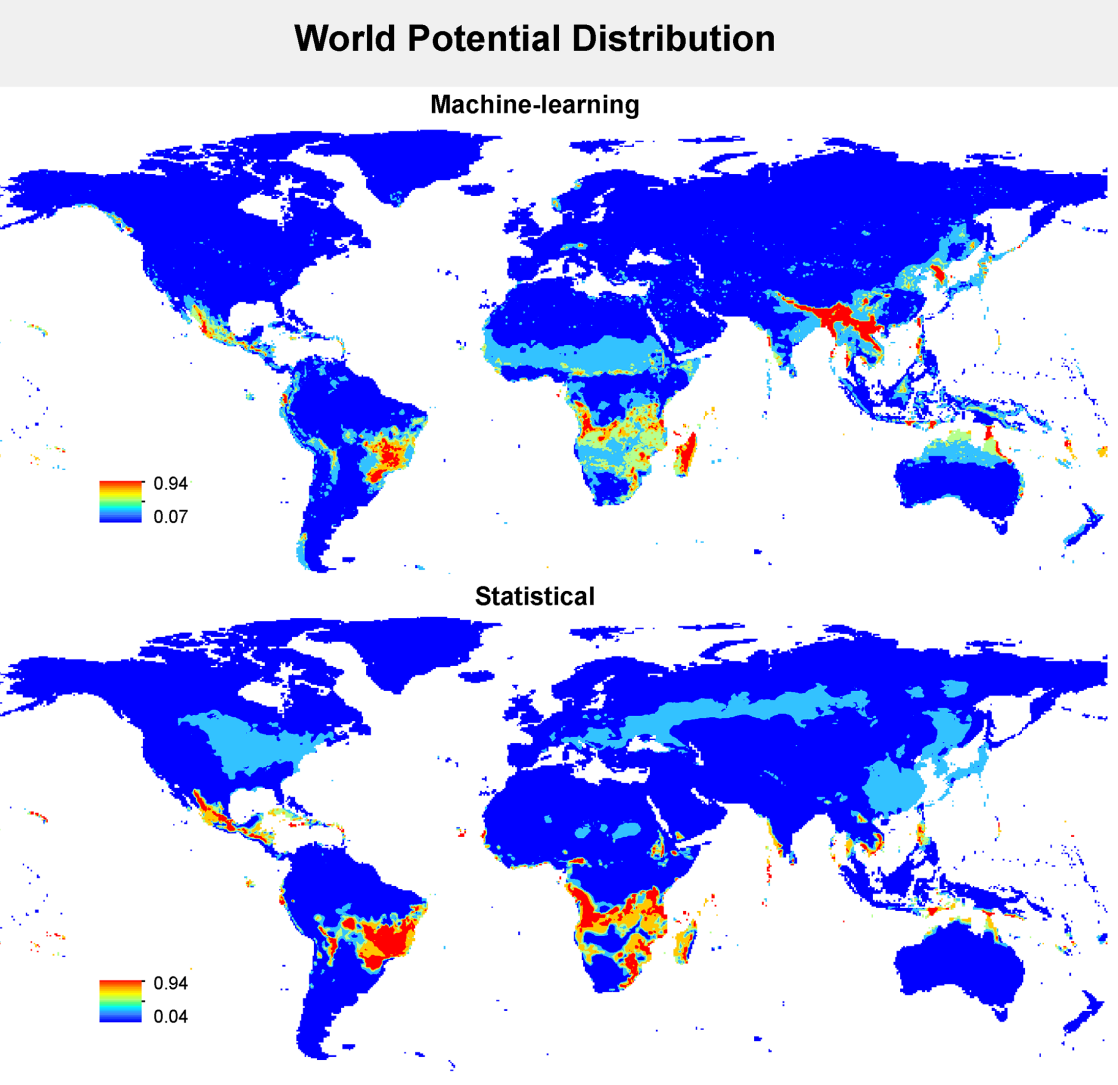
Current projection for dry root rot distribution in common beans in the world according to statistical and machining-learning methods. The model performed in Brazil was used to predict the dry root rot distribution in different places in which the host crop is grown, such as Central America, North of USA, Europe, Africa, and Asia. The legend shows the climatic suitability: 1 is the most adequate, and 0 is the least suitable.

Although the predictions of the current disease distribution in Brazil were not identical between statistical and machine-learning models, both methods predicted high disease risk in the central, southeastern, and southern portions of the country (Fig 1). Statistical methods predicted larger distributional areas for disease occurrence compared with machine-learning methods. Similar pattern was found in worldwide maps of the current disease distribution. Areas predicted as climatically highly suitable for disease occurrence were quite convergent between statistical and machine-learning methods (Fig 2). Again, on a worldwide scale, statistical methods produced less stringent distribution predictions compared with machine-learning methods.

**Fig 3:**
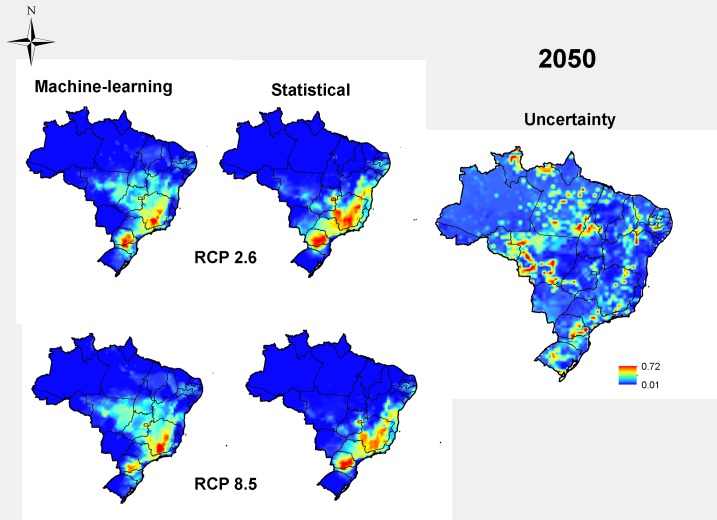
Future projection by 2050 for dry root rot in common bean in two RCP scenarios via a consensus between machine-learning and statistical methods, supported by distribution models based on inoculum densities of at least 3700 propagules/ gram of field soil in the current scenario, along with an estimate of uncertainty of projections. The legend shows the climatic suitability: 1 is the most adequate, and 0 is the not suitable.

**Fig 4:**
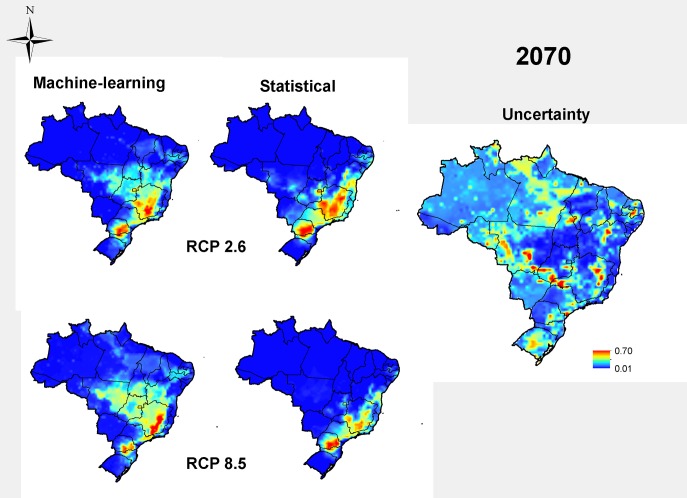
Future projection by 2070 for dry root rot in common bean in two RCP scenarios via a consensus between machine-learning and statistical methods supported by distribution models based on inoculum densities of at least 3700 propagules/ gram of field soil in the current scenario, along with an estimate of uncertainty of projections. The legend shows the climatic suitability: 1 is the most adequate, and 0 is the not suitable.

We considered two future scenarios according to two extreme emission rates of greenhouse gas (the “optimistic” RCP 2.6 and the “pessimistic” RCP 8.5), from the IPCC-AR5, estimated for the years of 2050 and 2070. In Brazil, an overall reduction in the suitable area for rot root occurrence in the common bean is expected in the future (Fig 3). In year 2050, a 49% reduction of the potential distribution is expected under the optimistic greenhouse gas emission rates, and up to 48% under the pessimistic climate change scenario. The high-climate-suitability area moved toward Southern Brazil, keeping disease occurrence outside of the Center-West Region of Brazil. That same reduction is observed in year 2070 projections (Fig 4). In 2070, for the RCP 2.6, reductions average 26% of the total area, and for the RCP 8.5, the reduction in the disease distribution area reached 41%. More details about the projections are in Table 1. The methods of estimation showed high values of TSS, being 75.5 to statistical and 92.3 to machine-learning. The machine-learning showed more precise models than statistical models. Most models had high overall accuracy (TSS>0.5), and bioclimatic variables presented different weights in estimation, according to the modelling method, with isothermality (up to 43.5%), maximum temperature of warmest month (up to 43.84%) and precipitation seasonality (up to 42.92%) the most important variables. Only isothermality showed congruence between Machine-Learning and statistical methods. (Table 2).

**Table 1:** Range of predictions of statistical and machine-learning methods within RCP scenarios in 2050 and 2070 for common bean dry root rot distribution in Brazil, compared with the current disease potential distribution.

Statistical				
AOGCM	2050		2070	
	RCP 26 (%)	RCP 85 (%)	RCP 26 (%)	RCP 85 (%)
MIROC5	-56	-52	-42	-62
CCSM4	-46	-44	-32	-68
HADGEM2	-61	-62	-57	-83
Average	-54	-53	-44	-71
Machine-Learning				
Miroc5	-3	-1	-20	-31
CCSM4	-2	-7	-12	-26
Hadgem2	-9	-6	-21	-32
Average (%)	-5	-5	-18	-30
Total average range (%)^1^	-49	-48	-26	-41

^1^ Difference between Machine-Learning and Statistical average range

**Table 2:** Relative importance of the bioclimatic variables by method of estimation weighted by TSS of models.

Climate Variable	Statistical (%)^1^	Machine learning (%)^2^
Isothermality	43.50	40.61
Max. Temperature of Warmest Month	31.13	43.84
Precipitation Seasonality	19.13	36.46
Precipitation of Warmest Quarter	17.63	42.92

^1^Weighted mean by TSS values each statistical modelling method.

^2^Weighted mean by TSS values each machine-learning modelling method.

The uncertainty analysis showed differences in climate suitability estimation by the method, AOGCMs, and climatic scenario. However, the greatest source of uncertainty was the modelling method (statistical and machine learning). In addition, uncertainty varied geographically in a splash-like pattern, and it concentrated in regions predicted as inadequate for disease occurrence. That splashed pattern was evident in both the years of 2070 and 2050. Moreover, the relative contribution attributed to each factor (i.e., variation ranking) did not change with the variation of fixed factors observed via eBLUEs (Table 3). The climate suitability of disease was lower in the scenario RCP 8.5 compared to RCP 2.6, which is expected avoid to RCP 8.5 to be pessimist, that is, shows a higher elevation of the average temperature. The low eBLUP values also show low interference with climate suitability as a response variable in 2050 and 2070. For each year, in 2050, the climatic favorability increased by 4.17 units above average, and in 2070, it decreased by 4.17 units above average (eBLUPs). This represents a low influence that only different years would explain about the climate suitability of the disease.

**Table 3:** The eBLUE values of standardized climatic favorability in a mixed model for uncertain analysis among estimate methods, AOGCMs, and scenarios as fixed factors, and “year” as a random factor (2050 and 2070).

Scenario	Method	AOGCM	eBLUE
RCP 2.6	Machine learning	CCSM 4	132.79
RCP 8.5	Machine learning	CCSM 4	128.88
RCP 2.6	Statistical	CCSM 4	132.04
RCP 8.5	Statistical	CCSM 4	101.95
RCP 2.6	Machine learning	HADGEM 2	129.53
RCP 8.5	Machine learning	HADGEM 2	128.53
RCP 2.6	Statistical	HADGEM 2	103.53
RCP 8.5	Statistical	HADGEM 2	83.97
RCP 2.6	Machine learning	MIROC 5	131.00
RCP 8.5	Machine learning	MIROC 5	129.08
RCP 2.6	Statistical	MIROC 5	122.08
RCP 8.5	Statistical	MIROC 5	106.16

## Discussion

Plant pathogens and their diseases will likely follow the climate-mediated distributional shifts on crops [4], thus creating a dynamic scenario in the management of plant disease epidemics in the face of climate change. Highlighting which regions are expected to be most at risk of disease is therefore crucial for disease management policies [6], and therefore, maps that anticipate disease risks can potentially increase efficiency and reduce the costs of disease management strategies in the future [2]. In this study, we modelled the distribution of common bean dry root rot, which stems from the FSSC. To do so, we used a spatial proxy for disease occurrence, represented by the inoculum density most correlated to disease distribution. An intermediate inoculum density (3700 propagules per soil gram) was the best proxy of disease occurrence, perhaps a more representative estimate of infestation by the FSSC in Brazilian common bean fields. The potential distribution of the disease in both the present and future times was also remarkably convergent to the predicted tropical areas (Central and South American and African continent) as highly suitable for common bean crops [54]. Even though records about the spatial distribution of dry root rot are scarce in the literature, our results regarding the world distribution of the disease also match the disease reports in countries such as Kenya [56], Rwanda [56], Burundi [56], Zaire [56], Mexico (such as the Aguascalientes, Veracruz, and Guanajuato states) [57] and the USA (such as Minnesota and North Dakota) [58]. All reports highlighted the importance of dry root rot as a result of relevant yield losses and difficulties of control.

In this study, the dry root rot distribution in the common bean was linked to all inoculum densities, but the intermediate threshold was the best spatial proxy for disease occurrence. The correlation between the inoculum threshold and disease occurrence therefore exhibited an intermediate saturation baseline [59]. The density-dependent relationships of the population growth of soilborne pathogens may be the cause of such patterns in epidemiology [60] as well as interspecific relationships in the soil community [61]. Soilborne pathogens may be endemic and cause diseases in natural ecosystems, but their main nutrient resource, the host, is obviously a crucial driver of their distribution. However, pathogen populations are also dependent on a certain within-host density that does not overcome the host’s carrying capacity, to avoid its complete depletion [59]. This is the case of FSSC × common bean, which results in stunted, low-yield plants and rarely in plant death. A host supportability therefore exists so that the mechanisms of intra and inter competition might regulate the abundance of soilborne pathogens on a small scale [62].

That intermediate aggressiveness paradigm has been observed on several root rot pathosystems in different regions of world. For example, the inoculum densities of *Verticillium dahliae*, *Cylindrocladium clotalariae*, *Rhizoctonia oryzae*, *F*. *oxysporum* f.sp. *gossypii* and *phaseoli*, and incidences of wilt in cotton [63], root rot on peanuts [64], root rot on barley [65], and wilt incidence in cotton [66] and the common bean [17] respectively are all density-dependently modulated, thus suggesting a general pattern. The intermediary aggressiveness of pathogens may be a result of evolutionary mechanisms that regulate density-dependent populations to maintain their fitness in the long term [59].

Using the intermediate inoculum density as a spatial proxy for disease occurrence allowed us to project the distribution of dry root rot in the future scenarios of climate change. Its occurrence will be probably reduced overall in the future based on the reduction of areas that are climatically suitable for the disease. In Brazil, the common bean dry root rot distribution is expected to shift toward the southern and southwestern regions of the country, which is more representative of small-scale farming than the Center-West Brazil is. On the other hand, regions that nowadays respond to high yields of the common bean, such as the Brazilian Center-West, will probably lose climatic suitability in the future.

Other diseases are also projected to move toward colder regions due to climate change [67]. Crop diseases stemming from soilborne pathogens, such as *F*. *nivale*, *F*. *culmorum*, *Macrophomina phaseolina*, *Sclerotinia minor*, and *Pythium ultimum*, on the European continent are all predicted to migrate in the direction of cooler areas [4]. Such results suggest that policies on disease management might benefit from focusing on the areas predicted to gain agricultural relevance, such as in the Brazilian scenario. Meanwhile, the breeding of resistant cultivars and the development of other environmentally friendly practices for disease integrated management plans may anticipate such changes and reduce food security risks.

We found a strong convergence of the statistical modelling in the tropical areas predicted as highly suitable for disease occurrence and the suitable areas for common bean cropping from an independent work. Besides predicting similar areas throughout the world, the same shrinkage pattern we found here was projected for common beans, from projections of physiological mechanistic models [54]. Projections in temperate regions, however, showed less convergence and were not so clear, probably because our dataset did not encompass temperate environmental conditions, underrating disease occurrence in North American [58] and Canadian regions, where dry root rot is relevant [68]. So far, no comparative data exist between disease severity between tropical and temperate regions. However, the European region shows low importance for the common bean because other crops are more important there, such as wheat [69]. At least in the tropics, both the host and the pathogen seem to share the same environmental requirements and constraints.

The integration of an environment conducive to pathogen infection and pathogen infectivity, via pathogen-host co-evolution mechanisms, may be one of the main drivers of disease dynamic distribution in the face of climate change [70]. Climate seasonality also affects soilborne pathogens’ density. Consequently, disease occurrence may shift according to climate seasonality. Temperature and precipitation regimes are also important drivers of soil pathogen distribution. Understanding the resilience of such soilborne pathogens against abiotic stress can potentially guide disease management plans [4].

The overlap of the predicted distribution of the disease and host therefore suggests that climate-mediated ecological and evolutionary mechanisms are the likely drivers of the distribution of common bean dry root rot. Indeed, co-evolutionary mechanisms between natural pathogen populations and their hosts [71], coupled with pathogen evolution in agricultural landscapes [72], have been attributed as the main drivers of disease occurrence. Although a discussion on host and pathogen co-evolution is not the purpose here, our results indicate the high climatic favorability of dry root rot in the Mesoamerican region, a hot spot of diversity where wild *P*. *vulgaris* has its origin [73]. This is an area where the relevance of dry root rot and the diversity of Fusarium species is well documented [57].

Climate change will affect the distribution of the common bean [54]. Here, we found that the distribution of common bean dry root rot will probably follow the same standard. In such cases, disclosing which regions of higher disease risk will drive greater attention to crop and disease management. Maps of disease risk are therefore crucial if we are to prevent economic losses, stemming from climate change, in regions that currently do not exhibit high pressure of diseases [6,74]. Here, we provide the first worldwide maps on the potential distribution of the common bean dry root rot. The statistical-based map has straightforward applications for disease management, especially developing countries from tropical regions, such as Latin America and the African continent [75]. By anticipating maps of disease risk, our work may help with the prioritization of financial and technological resources toward high-risk areas, thus possibly reducing the costs of disease management in the future [2]. Moreover, our approach may the adjusted to other pathosystems to predict disease occurrence and improve food security.

## Supporting information

S1 Fig Current and future projections of dry root rot of common beans according to the inoculum thresholds of 1200, 3700 and 4500 propugules per soil gram (PPG). (TIF)

S1 Table Dry root rot occurrence in Brazilian municipalities according to disease records in common bean fields, by several publications. Scientific papers, short communications, and technical documents were selected according to their reports of the disease outbreaks in different sampling sites.
(DOCX)

S2 Table Effect of the climate change on disease proxies according to statistical method. (DOCX)
