# Supplementary figures and images for: Potential worldwide distribution of Fusarium dry root rot in common beans based on the optimal environment for disease occurrence
Source: PLoS One. 2017 Nov 6;12(11):e0187770. doi: 10.1371/journal.pone.0187770 (PMC5673228; doi:10.1371/journal.pone.0187770)

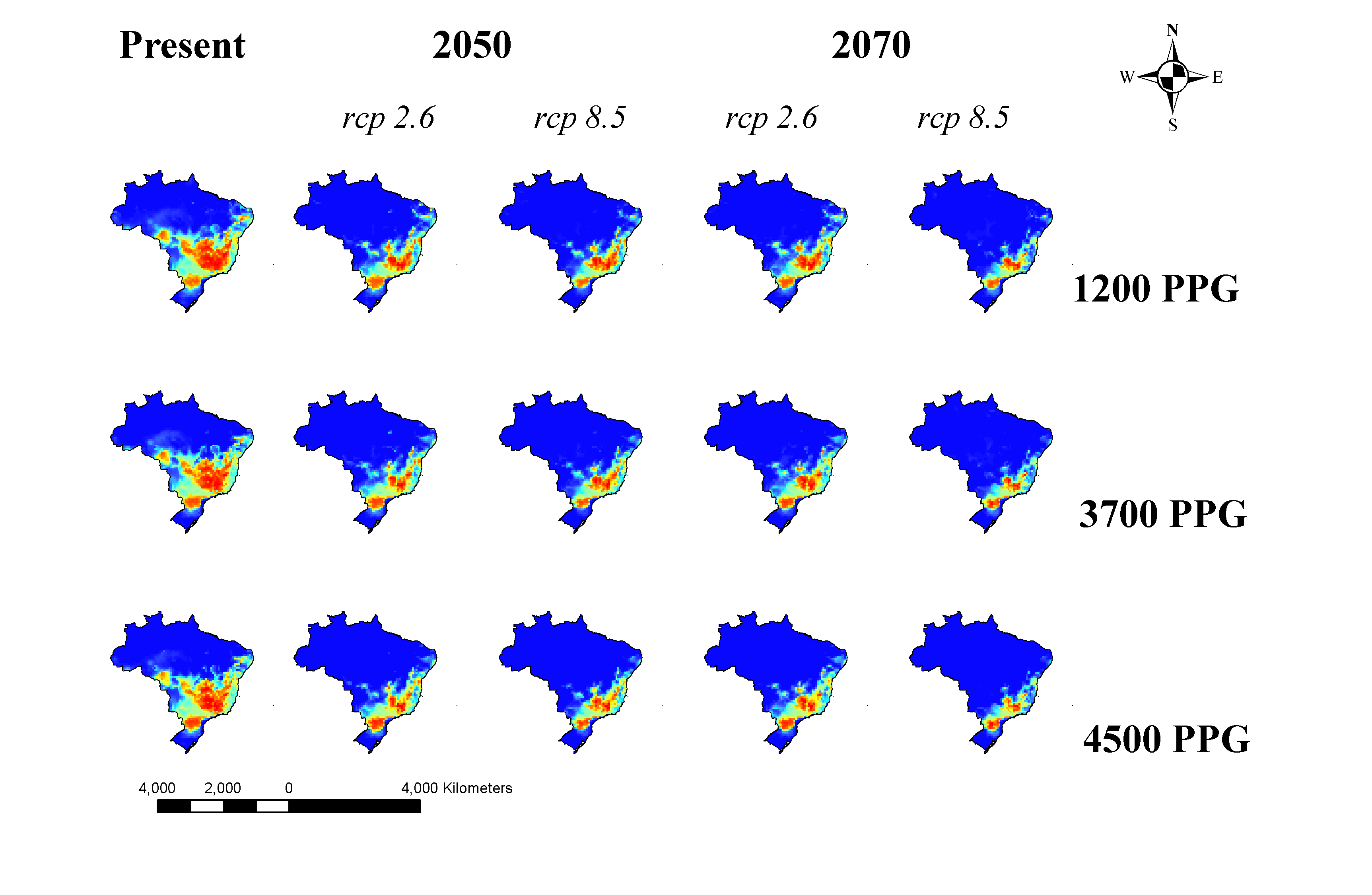

Supplement: S1 Fig — (TIF) [file pone.0187770.s001.tif]
