# Supplementary material for: Potential worldwide distribution of Fusarium dry root rot in common beans based on the optimal environment for disease occurrence
Source: PLoS One. 2017 Nov 6;12(11):e0187770. doi: 10.1371/journal.pone.0187770 (PMC5673228; doi:10.1371/journal.pone.0187770)
Supplement: S1 Table — Scientific papers, short communications, and technical documents were selected according to their reports of the disease outbreaks in different sampling sites. (DOCX) [file pone.0187770.s002.docx]

**Supporting information**

**S1 Table. Dry root rot occurrence in Brazilian municipalities according to disease records in common bean fields, by several publications. Scientific papers, short communications, and technical documents were selected according to their reports of the disease outbreaks in different sampling sites**

| **City** | **State** | **Samples** | **Latitude (UTM)** | **Longitude (UTM)** | **Zone** | **References** |
| --- | --- | --- | --- | --- | --- | --- |
| Goiania | Goias | roots | 686626 | 8156502 | 22k - WGS84 | [1] |
| Goiania | Goias | roots | 686626 | 8156502 | 22k - WGS84 | [1] |
| Acreuna | Goias | roots | 566180 | 8076547 | 22k - WGS84 | [1] |
| Silvania | Goias | roots | 755154 | 8156652 | 22k - WGS84 | [1] |
| Silvania | Goias | roots | 755154 | 8156652 | 22k - WGS84 | [2] |
| Silvania | Goias | roots | 755154 | 8156652 | 22k - WGS84 | [1] |
| Unai | Minas Gerais | roots | 296611 | 8189860 | 22k - WGS84 | [1] |
| Unai | Minas Gerais | roots | 296611 | 8189860 | 22k - WGS84 | [1] |
| Santo Antônio de Goias | Goias | roots | 680307 | 8176784 | 23K - WGS84 | [1] |
| Santo Antônio de Goias | Goias | roots | 680307 | 8176784 | 23K - WGS84 | [1] |
| Santo Antônio de Goias | Goias | roots | 680307 | 8176784 | 23K - WGS84 | [1] |
| Taquarituba | São Paulo | roots | 679274 | 7396403 | 23K - WGS84 | [1] |
| Formosa | Goias | soil | 249667 | 8280918 | 23L - WGS84 | [3] |
| Oratórios | Minas Gerais | soil | 728933 | 7739311 | 23K -WGS84 | [4] |
| Patos de Minas | Minas Gerais | soil | 339841 | 7945091 | 23K - WGS84 | [5] |
| Guaira | São Paulo | Soil/plant | 780811 | 7751063 | 22k - WGS84 | [6] |
| **City** | **State** | **Samples** | **Latitude (UTM)** | **Longitude (UTM)** | **Zone** | **References** |
| Castro | Paraná | soil | 599876 | 7257852 | 22J - WGS84 | [7] |
| Cristalina | Goias | soil | 221349 | 8144177 | 23K - WGS84 | [8] |
| Cristalina | Goias | soil | 221349 | 8144177 | 23K - WGS84 | [8] |
| Cristalina | Goias | soil | 221349 | 8144177 | 23K - WGS84 | [8] |
| Cristalina | Goias | soil | 221349 | 8144177 | 23K - WGS84 | [8] |
| Cristalina | Goias | soil | 221349 | 8144177 | 23K - WGS84 | [8] |
| Cristalina | Goias | soil | 221349 | 8144177 | 23K - WGS84 | [8] |
| Cristalina | Goias | soil | 221349 | 8144177 | 23K - WGS84 | [8] |
| Guarda Mor | Minas Gerais | soil | 277598 | 8033925 | 23K - WGS84 | [8] |
| Guarda Mor | Minas Gerais | soil | 277598 | 8033925 | 23K - WGS84 | [8] |
| Guarda Mor | Minas Gerais | soil | 277598 | 8033925 | 23K - WGS84 | [8] |
| Coromandel | Minas Gerais | soil | 267685 | 7956093 | 23K - WGS84 | [8] |
| Montes Claros de Goiás | Goias | soil | 457532 | 8230164 | 23K - WGS84 | [8] |
| Nova Ponte | Minas Gerais | soil | 213216 | 7874982 | 23K -WGS84 | [9] |
| Nova Ponte | Minas Gerais | soil | 213216 | 7874982 | 23K - WGS84 | [9] |
| Irai de Minas | Minas Gerais | soil | 240909 | 7899144 | 23K - WGS84 | [9] |
| Irai de Minas | Minas Gerais | soil | 240909 | 7899144 | 23K - WGS84 | [9] |
| Irai de Minas | Minas Gerais | soil | 240909 | 7899144 | 23K - WGS84 | [9] |
| Irai de Minas | Minas Gerais | soil | 240909 | 7899144 | 23K - WGS84 | [9] |
| Irai de Minas | Minas Gerais | soil | 240909 | 7899144 | 23K - WGS84 | [9] |
| Chapadao do Ceu | Goias | soil | 324025 | 7965551 | 22K - WGS84 | [9] |
| **City** | **State** | **Samples** | **Latitude (UTM)** | **Longitude (UTM)** | **Zone** | **References** |
| Chapadao do Sul | Mato Grosso do Sul | soil | 328975 | 7921198 | 22K - WGS84 | [9] |
| Chapadao do Sul | Mato Grosso do Sul | soil | 328975 | 7921198 | 22K - WGS84 | [9] |
| Chapadao do Sul | Mato Grosso do Sul | soil | 328975 | 7921198 | 22K - WGS84 | [9] |
| Londrina | Paraná | soil | 483357 | 7422153 | 22K - WGS84 | [9] |
| Vargem Grande do Sul | São Paulo | soil/plant | 304256 | 7584571 | 23K - WGS84 | [10] |
| São Gotardo | Minas Gerais | soil/plants | 389806 | 7864445 | 23K - WGS84 | [10] |

**References**

1. Oliveira VC De, Costa JLS, Compatibilidade JLS, Fusarium D. Compatibilidade Vegetativa de Nit-Mutantes de  *Fusarium solani*  Patogênicos e Não-Patogênicos ao Feijoeiro e à Soja. Fitopatol Bras. 2003; 89–92. doi:10.1590/S0100-41582003000100013

2. Costa R, Luis J, Costa S. Influência da densidade de inóculo de  *Fusarium solani*  f.sp. *phaseoli*  na severidade da podridão radicular seca do feijoeiro. Pesqui Agropecuária Trop. 2004;34: 89–92.

3. Prado TS, Brandão AS, Lobo-Junior M, Silveira PM. Distribuição de *Fusarium*  spp., *Trichoderma* spp. e *Rhizoctonia solani* no perfil do solo cultivado com feijoeiro comum em plantio direto e convencional. Doc IAC. 2008;85: 1043–1046.

4. Teixeira H, José T, Júnior DP, Vieira RF, Barreto M, Geovana C, et al. *Trichoderma* spp. decrease Fusarium root rot in common bean. Summa Phytopathol. 2012;38: 334–336.

5. Oliveira RM De, Vieira BS, Fagan EB. Interação entre diferentes teores de umidade do solo e patógenos do sistema radicular do feijoeiro. Cerrado Agrociências. 2014; 62–73.

6. Valarini PJ, Spadotto CA. Identificação de nichos de sobrevivência de fitopatógenos em áreas irrigadas de Guaíra, Sp. Pesqui Agropecu Bras. 1995;30: 1239–1243.

7. Valentini MHE, Ronzelli-Júnior P, Daros E, Pauletti V, Koehler HS. Épocas de manejo químico de coberturas de solo para o feijoeiro em plantio direto. Sci Agrar. 2001;2.

8. Ulhoa LA, Barbosa ET, Lobo-Junior M. Identificação, patogenicidade de isolados de  *Fusarium solani*  e severidade da podridão radicular no feijoeiro comum. 5^o^ Seminário Jovens Talentos. 2011. p. 53.

9. Costa SB, Campos VP, Menezes M. Fungos Associados a Cistos de  *Heterodera Glycines*  no Brasil. Nematol Bras. 1997;21: 31–37.

10. Silva FP. Estudos morfológicos e moleculares de *Fusarium oxysporum* f.sp. *vasinfectum*. 2013.
