# Supplementary material for: Potential worldwide distribution of Fusarium dry root rot in common beans based on the optimal environment for disease occurrence
Source: PLoS One. 2017 Nov 6;12(11):e0187770. doi: 10.1371/journal.pone.0187770 (PMC5673228; doi:10.1371/journal.pone.0187770)
Supplement: S2 Table — (DOCX) [file pone.0187770.s003.docx]

**Supporting information**

**S2 Table. Effect of the climate change on disease proxies according to statistical method**

| Proxy |  | AOGCM |  | 2050 | | |  | 2070 | | |
| --- | --- | --- | --- | --- | --- | --- | --- | --- | --- | --- |
|  |  |  |  | RCP 26 (%) |  | RCP 85 (%) |  | RCP 26 (%) |  | RCP 85 (%) |
| 1200 ppg |  | MIROC5 |  | -43 |  | -52 |  | -39 |  | -59 |
| 1200 ppg |  | CSSM4 |  | -29 |  | -53 |  | -27 |  | -66 |
| 1200 ppg |  | HADGEM2 |  | -57 |  | -69 |  | -53 |  | -80 |
| Average |  | - |  | -43 |  | -58 |  | -40 |  | -68 |
| 3700 ppg |  | MIROC5 |  | -56 |  | -52 |  | -42 |  | -62 |
| 3700 ppg |  | CSSM4 |  | -46 |  | -44 |  | -32 |  | -68 |
| 3700 ppg |  | HADGEM2 |  | -61 |  | -62 |  | -57 |  | -83 |
| Average |  | - |  | -54 |  | -53 |  | -44 |  | -71 |
| 4500 ppg |  | MIROC5 |  | -42 |  | -51 |  | -45 |  | -68 |
| 4500 ppg |  | CSSM4 |  | -35 |  | -58 |  | -39 |  | -76 |
| 4500 ppg |  | HADGEM2 |  | -60 |  | -70 |  | -65 |  | -86 |
| Average |  | - |  | -46 |  | -60 |  | -50 |  | -77 |
